# Structure of *Aspergillus flavus* populations associated with maize in Greece, Spain, and Serbia: Implications for aflatoxin biocontrol on a regional scale

**DOI:** 10.1111/1758-2229.13249

**Published:** 2024-04-17

**Authors:** Mohamed Ali Ouadhene, Kenneth A. Callicott, Alejandro Ortega‐Beltran, Hillary L. Mehl, Peter J. Cotty, Paola Battilani

**Affiliations:** ^1^ Department of Sustainable Crop Production Università Cattolica del Sacro Cuore Piacenza Italy; ^2^ USDA‐ARS Tucson Arizona USA; ^3^ International Institute of Tropical Agriculture Ibadan Nigeria; ^4^ College of Food Science and Engineering Ocean University of China Qingdao China

## Abstract

*Aspergillus flavus* is the most frequently identified producer of aflatoxins. Non‐aflatoxigenic members of the *A. flavus* L strains are used in various continents as active ingredients of bioprotectants directed at preventing aflatoxin contamination by competitive displacement of aflatoxin producers. The current research examined the genetic diversity of *A. flavus* L strain across southern Europe to gain insights into the population structure and evolution of this species and to evaluate the prevalence of genotypes closely related to MUCL54911, the active ingredient of AF‐X1. A total of 2173L strain isolates recovered from maize collected across Greece, Spain, and Serbia in 2020 and 2021 were subjected to simple sequence repeat (SSR) genotyping. The analysis revealed high diversity within and among countries and dozens of haplotypes shared. Linkage disequilibrium analysis indicated asexual reproduction and clonal evolution of *A. flavus* L strain resident in Europe. Moreover, haplotypes closely related to MUCL54911 were found to belong to the same vegetative compatibility group (VCG) IT006 and were relatively common in all three countries. The results indicate that IT006 is endemic to southern Europe and may be utilized as an aflatoxin mitigation tool for maize across the region without concern for potential adverse impacts associated with the introduction of an exotic microorganism.

## Graphical abstract

The current research examined the genetic diversity of *A. flavus* L strain across southern Europe to evaluate the prevalence of genotypes closely related to MUCL54911, the active ingredient of AF‐X1. A total of 2173 isolates recovered from maize collected across Greece, Spain, and Serbia in 2020 and 2021 were subjected to simple sequence repeat (SSR) genotyping. The analysis revealed high diversity within and among countries and dozens of haplotypes shared. Linkage disequilibrium analysis indicated asexual reproduction and clonal evolution of *A. flavus* L strain resident in Europe. Haplotypes closely related to MUCL54911 were found to belong to the same vegetative compatibility group (VCG) IT006 and were relatively common in all the three countries. The results indicate that IT006 is endemic to southern Europe and may be utilized as an aflatoxin mitigation tool.

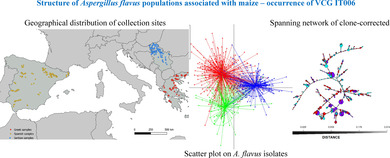

## INTRODUCTION

Maize (*Zea mays* L.) is one of the world's most important crops in terms of production, international trade, and provision of calories for livestock and humans, and it contributes to the socio‐economic balance in many countries (Wu, 2015). However, maize is susceptible to aflatoxin (AF) contamination from crop development through consumption (Palumbo et al., 2020; Strosnider et al., 2006). While aflatoxins frequently occur in tropical and subtropical regions, AF outbreaks were first reported in Europe in 2003 (Piva et al., 2006). AF contamination is currently a perennial problem in Europe due to climate change, particularly in maize‐growing regions of southern Europe including Italy, Spain, Greece, and Serbia (Battilani et al., 2016; Curtui et al., 2004; Moretti et al., 2019). AF concentrations in some years are sufficiently high to interfere with commercial use of the crop (Dobolyi et al., 2013; Levic et al., 2013; Udovicki et al., 2019). Aflatoxins (AFB1, AFB2, AFG1, AFG2, and AFM1) are potent mutagenic and carcinogenic mycotoxins that are naturally produced by several species in *Aspergillus* section *Flavi* (Janić Hajnal et al., 2017; JECFA, 2017). AFB1 is a highly potent liver carcinogen in humans and several domestic animal species; it is also immunotoxic and hepatotoxic and contributes to impaired productivity and reproductive efficiency in livestock (IARC, 2007; Kollia et al., 2017; Valencia‐Quintana et al., 2020).

*Aspergillus flavus*, the most commonly reported aflatoxin‐producer, is divided into two morphologically distinct strains, the L and S, with the widely distributed L strain fungi most frequently identified (Cotty, 1989; Cotty et al., 2008). L strain fungi have varying abilities to produce aflatoxins, with some genotypes unable to produce aflatoxins, while S strain fungi are potent aflatoxins producers typically found in hot, dry agroecosystems in the United States (Singh et al 2020). Fungi having similar morphology but belonging to different species have been reported relatively recently in the United States and various countries in sub‐Saharan Africa (Probst et al. 2012; 2014; Frisvad et al., 2019; Singh et al 2020) but rarely in Europe (Giorni et al., 2007; Perrone et al., 2014). In the current study, the L strain will be the main subject of study and hereafter *A. flavus* will refer only to the L strain.

*Aspergillus flavus* is well known as an abundant saprophyte and opportunistic pathogen and is ubiquitous in warm environments (Grubisha & Cotty, 2009; Horn, 2003; Klich, 2002). The life cycle of this predominantly asexual species is characterized by profuse production of haploid conidia (Islam et al., 2018; Papa, 1986). However, some studies have reported that sexual recombination can occur between *A. flavus* genotypes with different mating type loci (Horn et al., 2009; Moore et al., 2009). When considering natural populations, those that are present in the agroecosystem with no intentional modification by humans, both mating types, MAT1‐1 and MAT1‐2, co‐occur. However, the frequency of sexual reproduction in natural populations is less clear (Geiser et al., 1998; Grubisha & Cotty, 2009; Olarte et al., 2012; Pál et al., 2007; Ramirez‐prado et al., 2008).

Genetic diversity of *A. flavus*, considered both as a species and within the L strain, is high (Islam et al., 2018; Ortega‐Beltran et al., 2020), with many different genotypes that can produce AF at varying levels (Chang & Ehrlich, 2010). Populations of *A. flavus* are complex and still not fully described (Grubisha & Cotty, 2009). Isolates can be segregated into many vegetative compatibility groups (VCGs) on the basis of complementation between nitrate non‐utilizing auxotrophs (Bayman & Cotty, 1991, 1993). Genetic information can be exchanged within a VCG through an asexual recombination process referred to as the parasexual cycle (Ehrlich et al., 2007; Grubisha & Cotty, 2015; Leslie, 1993; Papa, 1986) that includes hyphal fusions between fungi with identical heterokaryon incompatibility alleles. Characterizing genetic diversity within *A. flavus* allowed the identification of VCGs containing exclusively non‐aflatoxigenic strains (Mehl et al., 2012). Naturally occurring, native non‐aflatoxigenic strains have been used for decades to displace aflatoxin producers through a competitive exclusion mechanism (Bayman & Cotty, 1993; Cotty et al., 2007; Mehl et al., 2012), a form of biocontrol that is highly effective at reducing aflatoxin contamination in the United States, Africa, and Europe (Italy) (Cotty, 1994a, 1994b; Dorner & Lamb, 2006; Mauro et al., 2018; Moral et al., 2020; Ojiambo et al., 2018). Non‐aflatoxigenic genotypes of *A. flavus* are applied to soil on a grain carrier that supports growth, sporulation, and dispersal of the biocontrol fungus to the crop via wind and insects, thereby displacing aflatoxin‐producing fungi (Bock et al., 2004; Mehl et al., 2012). Though the grain carrier in the formulated product gives biocontrol strains the advantage of a readily available food source, the long‐term efficacy of aflatoxin biocontrol relies on the ability of applied non‐aflatoxigenic genotype to competitively exclude aflatoxin producers in soil and on crops. Assessment of genetic diversity in *A. flavus* populations allows for identification of non‐aflatoxigenic *A. flavus* VCGs particularly well adapted to target agroecosystems (Islam et al., 2020). Identification of native non‐aflatoxigenic VCGs with potential use in aflatoxin biocontrol programs allows avoiding the use of exotic strains, which may have potentially negative ecological effects if introduced into target agroecosystems (Mehl et al., 2012; Probst et al., 2011).

Aflatoxin contamination of crops is almost entirely due to several species in *Aspergillus* section *Flavi* (Frisvad et al., 2019; Houbraken et al., 2020), a taxonomic subgroup of genus *Aspergillus* that includes over 30 species, with the most agriculturally important aflatoxin producers being those most closely related to *A. flavus*. In Europe, investigations into compositions of *Aspergillus* section *Flavi* communities have provided useful information on the relative role of key species in the contamination process as well as the ability of specific fungi to produce sclerotia and AF (Giorni et al., 2007). Work on distributions of *A. flavus* L strain fungi in maize growing regions of Italy identified non‐aflatoxigenic *A. flavus* MUCL54911, an effective biocontrol active ingredient. In laboratory and fields trials, a product utilizing MUCL54911 reduced aflatoxin contamination by over 90% (Mauro et al., 2013, 2018). The product utilizing MUCL54911 is currently commercialized under the name AF‐X1 (Anonymous, 2022a, 2022b).

Despite studies on diversity of *A. flavus* resident in several countries, little information is available on *A. flavus* L strain population structure in Europe (Gallo et al., 2012; Perrone et al., 2014). Prior studies developed culture‐based and DNA‐based methods useful to study *A. flavus* diversity. These have been used to determine the predominant causal agent of AF contamination in various crops and to optimize selection of non‐aflatoxigenic genotypes in target agroecosystems (Mehl et al., 2012; Alejandro Ortega‐Beltran et al., 2020; Vlajkov et al., 2021). Vegetative compatibility analysis (VCA), which uses pairs of complementary nitrate non‐utilizing auxotrophs to group *A. flavus* isolates into specific VCGs, has been used to both characterize populations of *A. flavus* (Bayman & Cotty, 1991) and define non‐aflatoxigenic *A. flavus* active ingredients of biocontrol products (Cotty, 1994a, 1994b; Ehrlich & Cotty, 2004). Although accurate, VCA is time‐consuming and laborious (Das et al., 2008; Sweany et al., 2011). Consequently, several molecular methods have been developed to characterize *A. flavus* isolates more rapidly. To identify non‐aflatoxigenic isolates, cluster amplification pattern markers are used to monitor large deletions in the aflatoxin biosynthesis gene cluster of *A. flavus* through multiplex PCR (Callicott & Cotty, 2015; Vlajkov et al., 2021). In addition, to characterize *A. flavus* populations on a finer scale, detect diversity within VCGs, and contrast competitiveness and adaptability among specific genotypes of interest, many typing schemes using simple sequence repeats (SSR) or inter simple sequence repeats (ISSRs) have been developed (Grubisha & Cotty, 2010; Hadrich et al., 2010; Hatti et al., 2010; Molo et al., 2022; Sweany et al., 2011; Tran‐Dinh & Carter, 2000; Wang et al., 2012).

The current study examined the population structure and genetic diversity of *A. flavus* resident in maize growing areas in three European countries considered hotspots for aflatoxin contamination: Greece, Spain, and Serbia (Battilani et al., 2016; Koutsias et al., 2021; Leggieri et al., 2021; Van der Fels‐Klerx et al., 2019). Seventeen SSR markers, developed by Grubisha and Cotty (2009) and used to characterize *A. flavus* populations in Africa and North America (Grubisha & Cotty, 2010; Islam et al., 2018, 2020; Ortega‐Beltran et al., 2016), were applied to European populations for the first time in order to (I) study genetic diversity among and within countries, (II) obtain insight into divergence of *A. flavus* populations and distribution of common haplotypes among the three countries, and (III) investigate the frequency and distribution of isolates belonging to the same VCG to which MUCL54911, the active ingredient of AF‐X1, belongs in order to evaluate the potential extension of regulatory approval for use of AF‐X1 beyond Italy, the country from which the active ingredient was initially isolated. Large scale use of AF‐X1 in the three target countries can allow producing maize with reduced aflatoxin content for reduced densities of toxigenic fungi in the environment, and health, trade, and economic benefits.

## EXPERIMENTAL PROCEDURES

### Maize sample collections

**FIGURE 1:**
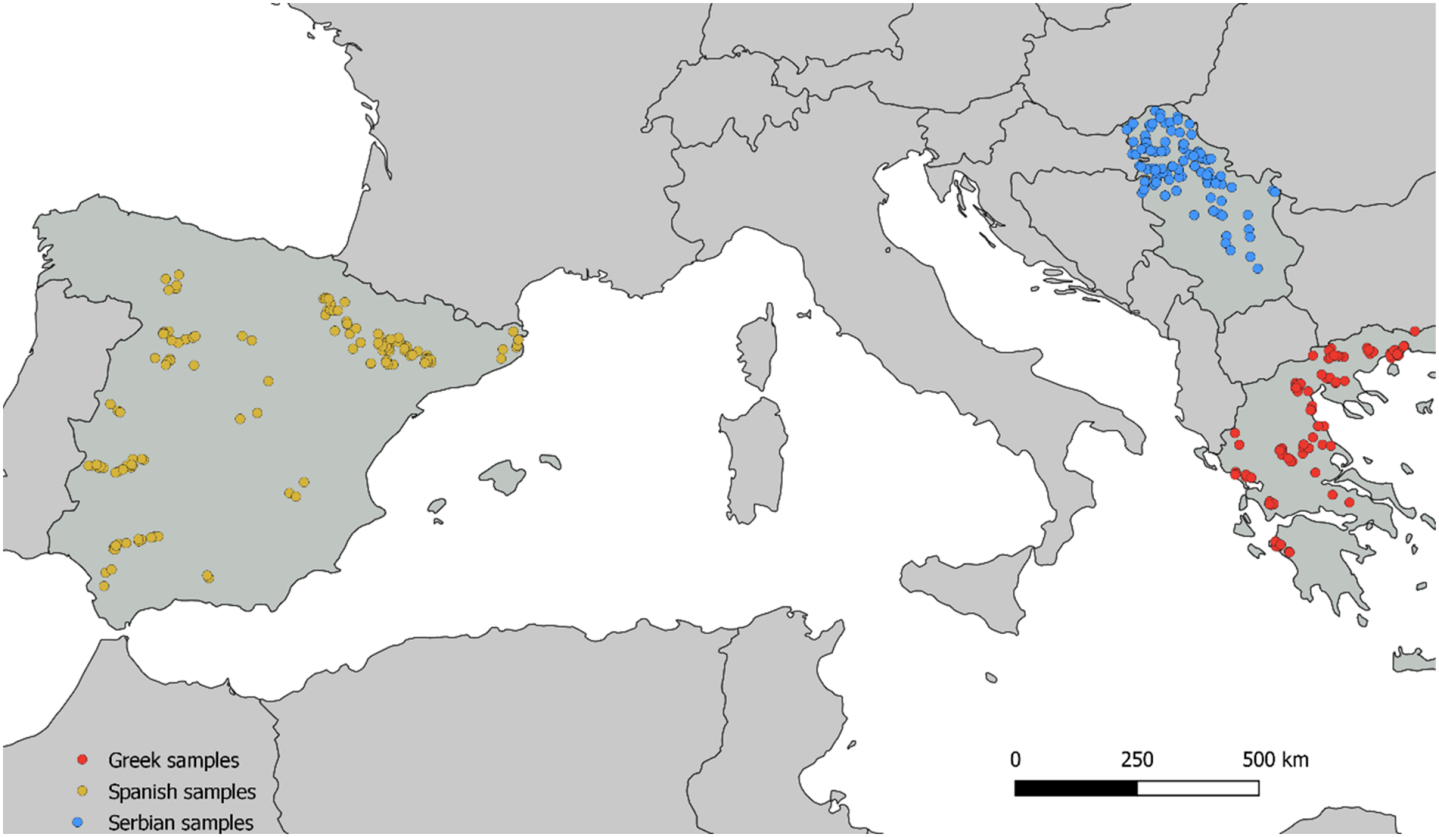
Geographical distribution of collection sites for samples from Greece (128), Spain (153) and Serbia (165) during maize harvest in either 2020 or 2021.

Grain samples were collected in areas known to have periodic *A. flavus* contamination across Greece (*n* = 128), Spain (*n* = 153), and Serbia (*n* = 165) (Figure 1). The grain was sampled either from the combine at harvest or upon receipt at an elevator before the drying process. Grain collection was initiated in late August 2020 and early September 2021. Each sample consisted of 30 sub‐samples (about 100 g of kernels, 3 kg total). After drying, samples were stored at 0–5°C and shipped to Italy within 3 days. Grain samples were ground, mixed to homogenize, partitioned into 2 aliquots of ~100 g, and stored at 5°C until processing for mycotoxin analysis and fungal isolation.

### Mycotoxins analysis

For each sample, 5 g flour was mixed with 20 mL acetonitrile/water/formic acid solution (59:20:1), vortexed for 30 min at 2500 rpm, and passed through a FPTE 0.20 μm filter. Mycotoxins were separated by loading 7 μL of the extract into an Ultimate HPLC machine (Thermo Scientific, Milford, MA, USA). Mycotoxins were identified with a calibrated Liquid Chromatography‐Mass Spectrometer (LC–MS) coupled with a Q Exactive Focus Orbitrap (Thermo Scientific). Aflatoxin concentrations were reported as the sum of AFB1, AFB2, AFG1, and AFG2. The limits of detection and quantification were 0.9 and 2.71 μg/kg, respectively.

### Aspergillus flavus isolation

Maize flour was serially diluted and plated on modified Rose Bengal agar (3 g sucrose, 3 g NaNO_3_, 0.75 g KH_2_PO_4_, 0.25 g K_2_HPO_4_, 0.5 g MgSO_4_·7H_2_O, 0.5 g KCl, 10 g NaCl, 1 mL of micronutrients, 0.025 g Rose Bengal, 0.05 g chloramphenicol, 0.05 g streptomycin, 0.01 g dichloran, 20 g Bacto agar, 1 L water) (Cotty, 1994a, 1994b). After 3–5 days of incubation at 35°C in the dark (Cotty, 1994a, 1994b), colony forming units (CFU) were counted, both for *A. flavus* and for other fungal species. Plates with less than 10 colonies were selected to recover *A. flavus* isolates, with no more than three isolates taken from any plate and up to 15 isolates per sample/field. Serial dilution was repeated to get a suitable number of isolates per plate. Selected isolates were transferred to 5/2 Agar (5% V8 juice and 2% agar, pH 6.0) and incubated for 5–7 days at 31°C (Jaime‐garcia & Cotty, 2004). Single spore isolation was done on the total set of 2173 isolates of *A. flavus* recovered from Greece, Spain, and Serbia; all monosporic isolates were saved in water vials for further use (Mauro et al., 2013; Ortega‐Beltran & Cotty, 2018).

### DNA extraction

All single spore isolates were grown on 5/2 agar and incubated for 7 days at 31°C. Spores were harvested from colonies by swab, after which DNA was extracted following the protocol of Callicott and Cotty (2015). In brief, spores were lysed in a buffer containing detergent and EDTA using a combination of heat and agitation. DNA was then extracted using a standard ethanol‐ammonium acetate precipitation. DNA concentration was determined with a NanoDrop 2.0 spectrophotometer (ThermoFisher, Wilmington, DE, USA) and diluted to adjust the concentration to 5 ng/μL.

### SSR genotyping

Seventeen SSR loci, previously characterized by Grubisha and Cotty (2009), were examined in this study (AF28, AF13, AF43, AF22, AF31, AF53, AF34, AF42, AF8, AF16, AF54, AF17, AF11, AF66, AF64, AF63 and AF55). SSR analyses followed the protocol described by Islam et al. (2018). SSR amplicons were free of PCR artefacts and had single peaks in the expected size range per locus based on Grubisha and Cotty (2009). Amplifications were performed by USDA‐ARS in Tucson, AZ, amplified markers were separated on an ABI 3730 at the University of Arizona's Arizona Genetics Core in Tucson, and fragment sizes estimated using GeneMarker 2.6.4 (SoftGenetics LLC, State College, PA) at USDA‐ARS.

### Population genetic analyses

Two Italian isolates (MUCL54911 and MPVP A2321) were included in the SSR analyses as references. The non‐aflatoxigenic strain MUCL54911 belongs to VCG IT006 (Mauro et al., 2013) and possesses the MAT1‐1 mating idiomorph. MPVP A2321 is non‐aflatoxigenic and has the MAT1‐2 idiomorph (Mauro et al., 2018).

For population analyses, incomplete SSR genotypes were excluded. The remaining SSR data from 2011 isolates out of 2173 and the two reference isolates (MUCL54911 and MPVP A2321) were processed with GENODIVE 3.06 (Meirmans, 2020). The R package poppr (Kamvar et al., 2014, 2015) was used to generate a minimum spanning network from clone‐corrected data using Bruvo distances (Bruvo et al., 2004). SplitsTree 4.8 (Huson & Bryant, 2006) was used to generate neighbour net trees using the Cavalli‐Sforza chord distance matrix generated by GENODIVE, following the protocol described by Ortega‐Beltran et al. (2020). GENODIVE 3.06 was also used to identify both shared and closely related multilocus genotypes among the three countries using a selected threshold among genetic distances. Haplotypes closely related to reference isolates were identified.

All isolates of *A. flavus* were sorted by province and country. Where sample size was less than 10 individuals, two or more adjacent provinces were combined to produce more reliable estimates of genetic diversity. GenAlEx version 6.5 (Peakall and Smouse, 2006) was used to evaluate number of alleles, number of private alleles and haploid genetic diversity (H), and to produce a principal coordinate analysis (PCoA) based on a pairwise genetic distance matrix (Peakall & Smouse, 2012).

Haplotype‐analysis version 1.05 (Eliades & Eliades, 2009) was used to identify multilocus SSR haplotypes (genotypes), their frequency within and among the populations, the number of private haplotypes (P_h_), the number of different haplotypes observed (Nh, individual population contribution to genetic diversity within populations (H_S_(j)), and individual population contribution to the total diversity among populations (D_ST_(j)) (Finkeldey & Murillo, 1999).

To determine the genetic relationships among countries, DAPC was generated by the *adegenet* package (Jombart, 2008; Jombart & Ahmed, 2011) in R software. This clustering analysis method was used for performing the first PCA, followed by a discriminant analysis on the PCA scores. The function *find. cluster*s in R was used to determine the right number of retained principle components for the DAPC analysis (Jombart & Sébastien Devillard, 2010). G'_ST_ (Hedrick, 2005) was used to determine the standardised genetic differentiation among alleles and was obtained from mmod package (Winter, 2012) in R. The *poppr* package (Kamvar et al., 2014) in R was used to evaluate the evenness of genotype frequency within groups based on the E_5_ calculation (Grünwald et al., 2003).

Linkage disequilibrium analysis was used to estimate the degree of clonality within populations. Multilocus genotypic LD within each country was calculated after clone correction by the *poppr* package using the unbiased estimator r¯_d_ (Agapow & Burt, 2001). Analysis of Molecular Variance (AMOVA; Excoffier et al., 2005) using *poppr* was performed to estimate population differentiation.

### Vegetative compatibility analysis (VCA)

Haplotypes from Greece, Spain, and Serbia that were closely related to MUCL54911, as revealed by the neighbour net tree analysis, were evaluated for membership in VCG IT006. To determine whether a haplotype belongs to VCG IT006, isolates from the selected haplotype were subjected to VCA using previously generated IT006 tester pair mutants (*cnx* and *niaD*), following the protocol described previously (Das et al., 2008).

## RESULTS

### Aflatoxin contamination, fungal densities, and number of isolates recovered

The respective percentages of grain samples positive for aflatoxin (AF) for 2020 and 2021 was 5% and 6% in Greece, 0% and 2% in Spain, and 0% and 35% in Serbia. In Greece, the maximum contamination was 52.6 μg/kg (mean 15.4 μg/kg in samples with detectable AF). In Serbia, one sample from 2021 contained 1148 μg/kg total AF. However, the mean AF concentration in samples with detectable aflatoxins in Serbia was 109.5 μg/kg. Only one sample from Spain was positive for AF with 2.64 μg/kg. In addition, the mean *A. flavus* CFU/g was similar in the three countries: 4.3 × 10^3^ in Greece, 1.6 × 10^3^ in Spain, and 7.8 × 10^3^ in Serbia. A total of 800 (Greece), 627 (Spain), and 758 (Serbia) *A. flavus* isolates were recovered from samples across these three countries (Figure 1) and used for analyses. Because some samples yielded too few *A. flavus* isolates to allow for robust analysis of population structure, samples that were collected from within approximately 10 km were pooled to represent ‘a priori populations’.

### Allelic and haplotypic diversity

SSR loci were found to be highly variable in amplicon size, with individual loci having between eight and 47 unique alleles (Table 1). High genetic diversity was detected among the 2011 isolates from the three countries. Haploid diversity (H) per locus ranged from 0.190 to 0.723. Evenness, which describes how similar in frequency the alleles for each SSR marker were, ranged from 0.40 to 0.79. Additionally, G'_ST_ of each marker varied between 0.134 and 0.666 (Table 1).

**TABLE 1:** Characteristics of 17 SSR markers from 2011 isolates of *A. flavus* recovered from maize sampled in Greece, Spain, and Serbia.

Locus name	Repeat motif and scaffold (Grubisha & Cotty, 2009)	Alleles^a^	Size range (bp)^b^	Diversity (H)^c^	Evenness^d^	G'_ST_ ^e^
AF8	(AAG)_16_/2911	35	147–267	0.722	0.64	0.664
AF11	(AAG)_12_/2504	38	103–281	0.683	0.54	0.486
AF13	(CTT)_9_/1866	23	115–200	0.669	0.67	0.627
AF16	(TTG)_10_/2541	22	161–393	0.437	0.54	0.450
AF17	(AGA)4 (AGG)_10_/1918	18	330–405	0.690	0.79	0.561
AF22	(TTTA)_8_/2911	12	144–208	0.537	0.63	0.487
AF28	(TTG)_11_/2504	15	110–161	0.455	0.63	0.460
AF31	(TTC)_31_/2634	32	290–415	0.588	0.41	0.478
AF34	(GTC)4 (GTT)_8_/2911	22	290–425	0.561	0.67	0.476
AF42	(TTC)_16_/2634	34	139–336	0.666	0.61	0.583
AF43	(GAG)_13_/2634	30	365–451	0.723	0.65	0.666
AF53	(TCT)_8_/1918	17	126–182	0.523	0.54	0.468
AF54	(ACAT)_8_/1918	9	145–192	0.190	0.40	0.267
AF55	(GT)_10_/1739	23	159–212	0.702	0.76	0.589
AF63	(AT)_7_/2856	8	121–137	0.217	0.40	0.134
AF64	(AC)_16_/2856	47	153–271	0.682	0.46	0.602
AF66	(AT)_12_/1569	14	198–279	0.589	0.78	0.543

^a^ Number of Alleles at the SSR locus.

^b^ Range of SSR size based on the variation at SSR repeat numbers across the isolates included in this study.

^c^ Per locus haploid genetic diversity (H) generated from the program GenAlEx6.5 (Peakall & Smouse, 2012).

^d^ Evenness obtained from the poppr package in R.

^e^ Standardised genetic differentiation (G'_ST_; Hedrick, 2005) obtained from mmod package in R.

**FIGURE 2:**
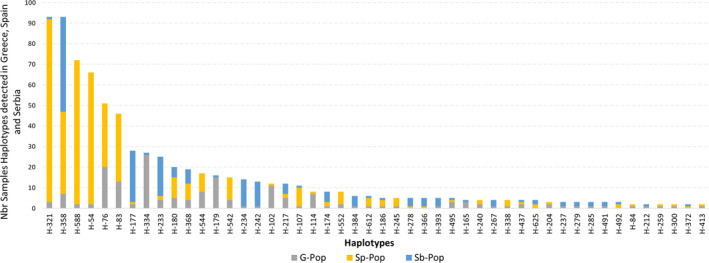
Distribution of the most frequent haplotypes shared among countries (G‐Pop, Greece; Sp‐Pop, Spain and Sb‐Pop, Serbia).

High haplotypic diversity was seen in Greece, Spain, and Serbia, with 363, 134, and 209 haplotypes (i.e., haploid multi‐locus genotypes) observed from 766, 574, and 671 isolates, respectively (Table 2). When the three countries were analysed together, 645 haplotypes overall were detected, illustrating the large number of haplotypes (61) present in more than one country (Figure 2). Evenness values, computed on haplotypes within each country (Table 2; Grünwald et al., 2003), were 0.440, 0.412, and 0.483 for Greece, Spain, and Serbia, respectively.

**TABLE 2:** Overview of the genetic diversity of *A. flavus* recovered from Greece, Spain, and Serbia during the 2020 and 2021 growing seasons.

Country	*N*	A priori populations	Ncc	Nh	P_h_	H_S_	H′	E_5_	${\bar{r}}_{D}$
Greece	766	23	511	363	304	0.906	1.29	0.440	0.229 (*p* = 0.001)
Spain	574	15	261	134	98	0.872	1.33	0.412	0.311 (*p* = 0.001)
Serbia	671	23	355	209	154	0.857	0.98	0.483	0.27 (*p* = 0.001)
Three countries	2011	61	1127	645	504	0.880	1.18	0.364	

*Note*: *N*, the total number of isolates; Ncc, number of isolates after clone correction by using poppr package in R (Kamvar et al., 2014); Nh, number of haplotypes; P_h_, number of private haplotypes; H_S_, within population genetic diversity from HAPLOTYPE‐ANALYSIS V1.04; H′ (Shannon, 1948), the Shannon information index calculated by GenAlex 6.503; E_5_ (Grünwald et al., 2003), evenness calculated using the poppr package in R; ${\bar{r}}_{D}$, index of association.

Clone correction by population illustrates the extent of haplotype diversity, with the number of haplotypes equating to 71%, 51%, and 59% of the clone corrected samples for Greece, Spain, and Serbia, respectively. The Spanish population had the most haplotypes shared among populations, and population‐based clone correction resulted in elimination of 55% of the original isolates. Similar corrections resulted in elimination of 33% for Greece and 47% for Serbia. After clone correction, gene diversity (H_S_) among countries ranged from 0.857 to 0.906, with Greece being the most diverse (Table 2). At the country level, estimation of the Shannon‐Wiener diversity index H (Table 2) among populations reflects high haplotypic diversity for all three countries (Greece = 1.29, Spain = 1.33 and Serbia = 0.98), with no dominance by any haplotype. Despite the number of isolates typed here, none of the countries have approached saturation based on a haplotype accumulation curve (Figure S1). Nevertheless, any remaining un‐sampled haplotypes are likely to be at very low frequency, and the following analyses of population structure should be approximately correct.

### Population structure, reproduction and evolution

**FIGURE 3:**
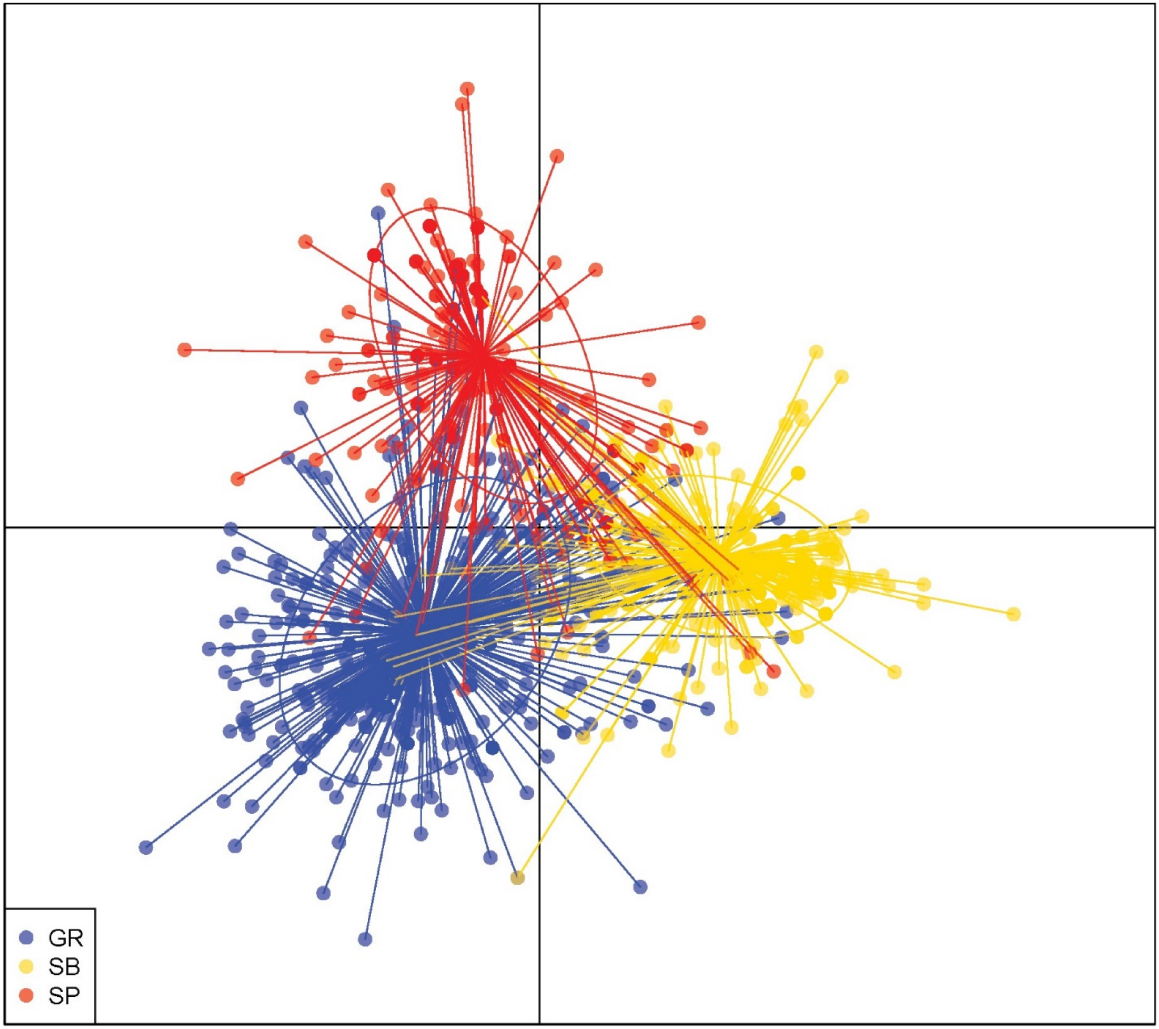
Scatter plot of three countries based on discriminant analysis of principal components (DAPC) on *Aspergillus flavus* isolates recovered from maize grain collected at harvest from locations (Figure 1) dispersed across three countries: Greece (red, G‐Pop = 766 isolates), Spain (Green, Sp‐Pop = 574 isolates) and Serbia (Blue, Sb‐Pop = 671 isolates).

Genetic variation overlapped extensively among the three countries, as shown by discriminant analysis of principal components (DAPC; Figure 3) and principal coordinate analysis (PCoA, Figure S2). DAPC also revealed different central tendencies for each country, indicating significant genetic differentiation even if these populations remain closely related. This genetic divergence can also be seen in an AMOVA performed on the entire dataset using countries and populations within countries as additional strata (Table 3; Figure S3).

**TABLE 3:** Analysis of molecular variance (AMOVA) of *Aspergillus flavus* isolates recovered from maize across Greece, Spain and Serbia.

Source of variation	df^a^	Sum sq^b^	Variance components	Percentage of variation	Φ^c^	*p*‐value^d^
Among countries	2	785.1572	0.234	1.92	0.04	0.001
Among a priori populations within countries	58	3015.116	0.367	2.96	0.03	0.001
Within a priori populations	1950	20601.32	11.81	95.11	0.01	0.001

^a^ Degree of freedom;

^b^ Sum of squares;

^c^ A measure of population divergence.

^d^ Significance was based on 10,200 permutations.

The results of linkage disequilibrium (LD) obtained after clone correction (Table 2, Figure S4), showed the unbiased indices of association, r¯_d_, are significantly (*p* < 0.01) greater than what is expected under sexual recombination in Greece, Spain, and Serbia (0.229, 0.311, and 0.270, respectively) and supportive of mutation driven divergence among lineages utilizing predominantly asexual reproduction.

### Genotype distribution and genetic differentiation

Although DAPC shows some differences among countries, many haplotypes were detected in more than one country (Figure 2). H‐588, H‐321, H‐358, H‐54, H‐76, H‐83, H‐177, H‐233, and H‐180 are the most frequent haplotypes, in decreasing order, found in more than one country and collectively represent more than 70% of all isolates before clone correction. Among the 645 haplotypes detected, 13 (2%) were detected in all three countries, and 35 (5%) haplotypes were detected in two of three countries. The remaining 597 (93%) haplotypes were detected in only one country. Number of haplotypes detected per country was higher in Greece (A = 363) and Serbia (A = 209) than in Spain (A = 134), reflecting the extensive clone correction for that country (Table 2). The two haplotypes detected in most samples were H76 (18 samples, with 7 in Greece and 11 in Spain) and H588 (17 samples, with 1 in Greece and 16 in Spain). Haplotypes detected in only a single population (private haplotypes) composed 78% of the 645 haplotypes detected among the 2011 isolates, 73% of those in Spain, 74% of those in Serbia, and 84% of those in Greece (Table 2). The analysis of molecular variance (AMOVA) results (Table 3) show both the separation among the three countries and the overwhelming diversity found within populations, representing over 95% of the total variation in the dataset. The variation among countries (1.92%) and among populations within countries (2.96%) are minute by comparison. These small levels of variation in higher levels of organization are still significant: Φ (calculated by AMOVA) increased at higher strata, ranging from 0.01 within populations to 0.03 among populations within countries to 0.04 among the countries themselves (Table 3). Increasing Φ indicates increasing coalescent times among or within populations with increasing genetic distance.

### Assignment of Aspergillus flavus haplotypes into VCG of MUCL54911

**FIGURE 4:**
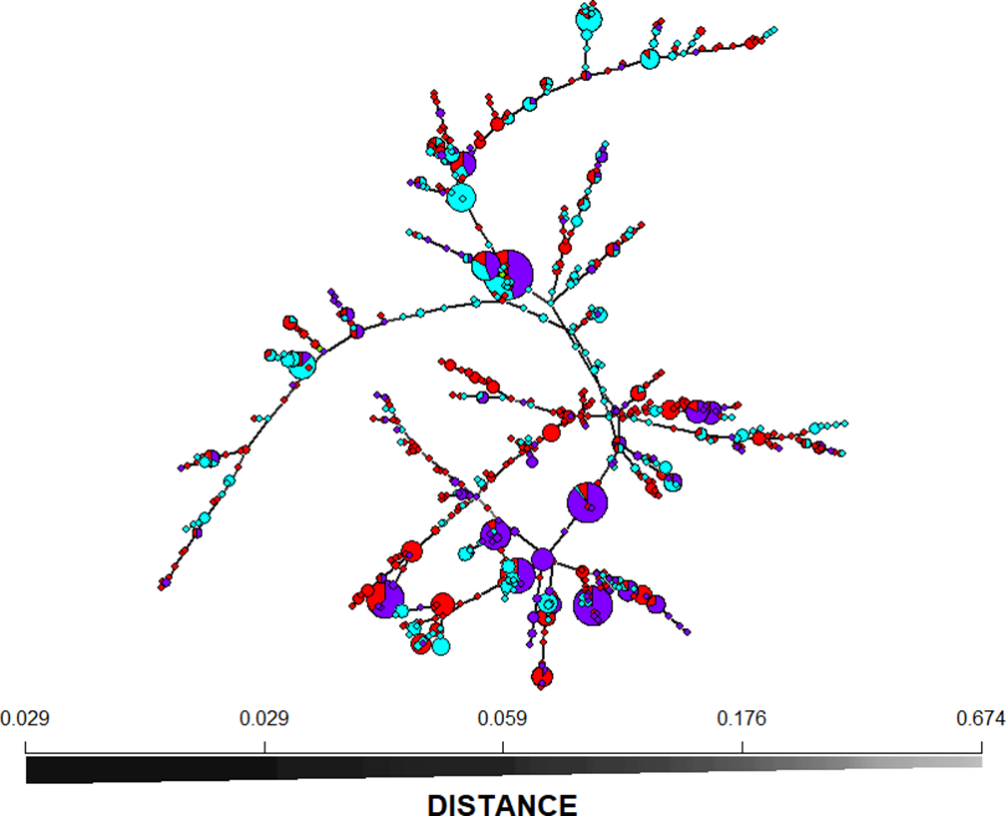
Minimum spanning network of clone‐corrected data for Greece, Serbia, Spain. The two Italian haplotypes were included for reference. Network created using the ‘poppr’ package in R. Haplotypes are coloured by where they were found (red = Greece, blue = Serbia, purple = Spain, and green = Italy). The size of circles is proportional to the number of times the haplotype was observed.

VCA revealed that isolates of 13 of the 14 haplotypes are most closely related to that of MUCL54911, the active ingredient of aflatoxin biocontrol product AF‐X1, belong to VCG IT006. A minimum spanning network (MSN) (Figure 4) and Nearest‐Neighbour networks (Figure S5) were used to display variation among haplotypes and to identify haplotypes closely related to the haplotype of isolate MUCL54911. All members of IT006 belong to a single branch of the MSN and are the only haplotypes along that branch. Although the frequency of IT006 was highest in Spain (8.9%), it was still well represented in Greece (2%) and Serbia (1.6%) (Table 4). In addition, the VCA revealed that an isolate with haplotype H‐373, differing from MUCL 54911 at 2 loci, does not belong to VCG IT006. As with MUCL54911, several haplotypes closely related to the haplotype of MPVP A2321, which is another Italian non‐aflatoxigenic haplotypes, were also found in multiple countries (data not shown).

**TABLE 4:** Haplotypes belonging to vegetative compatibility group (VCG) IT006, the VCG containing MUCL54911, the active ingredient of the aflatoxin biocontrol product AFX1.

Haplotype	Country	Isolates^a^ (#)	AF28^b^	AF13	AF43	AF22	AF31	AF53	AF34	AF42	AF8	AF16	AF54	AF17	AF11	AF66	AF64	AF63	AF55
MUCL54911	Italy	1	119	141	399	144	312	131	296	150	166	169	161	368	135	271	161	127	172
H‐358	Greece	7	119	141	399	144	312	131	296	150	166	169	161	368	135	271	161	127	180
	Spain	40																	
H‐368	Greece	4	119	141	399	144	312	131	301	150	166	169	161	368	135	271	161	127	180
	Serbia	7																	
	Spain	8																	
H‐360	Greece	1	119	141	399	144	312	131	296	150	166	169	161	368	135	271	163	127	180
H‐376	Greece	1	119	141	399	144	312	131	323	150	166	169	161	368	135	271	161	127	180
H‐367	Greece	1	119	141	399	144	312	131	301	150	166	169	161	368	132	271	161	127	180
H‐346	Greece	1	119	141	399	144	309	131	301	150	166	169	161	368	135	271	161	127	180
H‐375	Serbia	1	119	141	399	144	312	131	317	150	166	169	161	368	135	271	161	127	182
H‐359	Serbia	1	119	141	399	144	312	131	296	150	166	169	161	368	135	271	161	127	182
H‐378	Serbia	1	119	141	399	144	312	131	425	150	166	169	161	368	135	271	161	127	180
H‐278	Serbia	1	119	141	399	144	312	131	310	150	166	169	161	368	135	271	161	127	180
H‐370	Spain	1	119	141	399	144	312	131	307	153	166	169	161	368	135	271	161	127	180
H‐372	Spain	1	119	141	399	144	312	131	310	150	166	169	161	368	135	271	161	127	180
H‐374	Spain	1	119	141	399	144	312	131	317	150	166	169	161	368	135	271	161	127	180

^a^ Number isolates belonging to this haplotype. All members of this haplotype were tested by vegetative compatibility analysis (VCA) and found to belong to VCG IT006.

^b^ SSR loci and allele sizes. Alleles differing from MUCL‐54911 are shaded.

## DISCUSSION

When aflatoxin biocontrol products containing native non‐aflatoxigenic fungi as active ingredients are applied in the field using the correct dosage and at the right crop phenological stage, there is displacement of and reduced infection rates by aflatoxin producers (Mehl et al., 2012). This results in reduced aflatoxin contamination in the harvested crop. However, careful deployment of a non‐aflatoxigenic strain of *A. flavus* as a biocontrol agent for aflatoxin mitigation requires knowledge of the population structure of *A. flavus* including establishing that the non‐aflatoxigenic strain is endemic in the target country. Aflatoxin‐producing and non‐producing genotypes naturally occur in each country examined in the current study and are already resident in fields where aflatoxin control is needed. Use of native genotypes which are well adapted to the target agroecosystem should allow for more effective competition with aflatoxin producers and thus greater reductions in aflatoxin concentration in the target crop (Abbas et al., 2011; Cotty & Mellon, 2006; Moral et al., 2020; Peles et al., 2021). The reason for using native genotypes is twofold: first, native haplotypes have already shown they can survive under the local conditions, and second, using native haplotypes prevents the introduction of novel genetic types to the country and simplifies the process for gaining regulatory approval for biocontrol products. The present work provides new insights on populations of *A. flavus* resident in a large area spanning the European regions most susceptible to aflatoxin contamination (Spain, Greece, and Serbia; Figure 1). At the same time, this study describes the distribution of the genotype which comprises the active ingredient of the biocontrol product AF‐X1 belonging to VCG IT006, in southern Europe, providing a rationale for expanding the use of AF‐X1 throughout the region.

The current study had three major findings. First, the genotypic diversity of *A. flavus* within these countries across southern Europe is very high, as indicated by the haplotype accumulation curve (Figure S1). At the same time, these populations consisted of both locally restricted and widely dispersed common haplotypes among samples and among countries. Second, complete LD was observed in all countries (Table 2, Figure S4), supporting clonal evolution of *A. flavus* populations in southern Europe, as seen in other studies of natural populations of *A. flavus* (Grubisha & Cotty, 2015; Hadrich et al., 2013; Islam et al., 2018, 2020; Ortega‐Beltran et al., 2020; Picot et al., 2018). Finally, members of the VCG to which MUCL54911 belongs occur in all the sampled regions (Table 4), revealing a natural distribution of this biocontrol agent across southern Europe and opening the potential for use of MUCL54911 in the mitigation of aflatoxin contamination throughout this region. While the frequency of haplotypes belonging to IT006 are not high, even haplotypes observed at low frequency can be considered successful due to the very high variability found within fields and countries. The frequency observed here is similar to what has been seen for other biocontrol VCGs or SSR haplotypes prior to commercial application (Islam et al., 2020; Ortega‐Beltran et al., 2016).

Multiple measures of genetic diversity (Table 2) reflect a dataset comprised of a very large number of haplotypes, most of which occur at very low frequency (Figure 1). The number of haplotypes equals 57% of the total number of clone‐corrected isolates within the entire dataset, and private haplotypes (those seen in only one population) were 78% of all haplotypes. This variation in the dataset is reflected in haplotypic richness and diversity, and the large number of singleton haplotypes is reflected in Shannon's index and the evenness (Table 2). While most haplotypes were somewhat closely related, there is a small number of much more divergent haplotypes (Figure S2, S3). The importance of these divergent lineages to aflatoxin contamination in Europe is unknown. Considering the level of variation found among individuals, it is no surprise to find a great deal of variation among populations and countries. Gene diversity is quite high for each country individually and the dataset as a whole, and the skewed frequency distribution of haplotypes is again reflected in evenness and Shannon's index (Table 2). This pattern of diversity mirrors that seen in earlier population studies using VCA (e.g., Bayman & Cotty, 1993; Mauro et al., 2013) and also later studies using SSRs (e.g., Islam et al., 2018; Ortega‐Beltran & Cotty, 2018).

In the examined European *A. flavus* populations, nearly all genetic variation is found within populations, as shown by AMOVA (Table 3). There is low but significant diversity among populations and countries: the 3% of variation among populations within countries, and 2% of variation among countries are low relative to the amount of variation within populations. Nevertheless, there is significant population structure at all levels of the AMOVA, as indicated by Φ (Table 3; Figure S3). These results suggest local, mutation‐driven, clonal evolution as seen with the DAPC scatterplot. The data further suggests dispersal of both common and rare haplotypes across the sampled region of southern Europe (Figure 2). The very high haplotype diversity detected in each sampled country reduced the ability of this study to fully describe the distributions of many haplotypes. As a result, even with the large sample size of over 1100 clone‐corrected isolates, only a small minority (9%) of the 645 haplotypes were detected in more than one of the 61 a priori populations. However, the geographic range over which haplotypes detected in multiple populations were dispersed included the entire area sampled. Although diversity was so great that most (78%) haplotypes were detected only in one sample, dispersal of the haplotypes detected in multiple populations supports the three sampled countries having in common a single highly diverse community of *A. flavus* clonal lineages. Both rare (detected in only 2 of the 61 a priori populations) and relatively common *A. flavus* haplotypes were found dispersed across the southern portion of the European continent at various frequencies. This wide distribution suggests that non‐aflatoxigenic haplotypes found anywhere in Europe might be used as active ingredients in biocontrol products for use across the continent without concern about introducing a novel haplotype into a vulnerable habitat with detrimental impact (Islam et al., 2020; Probst et al., 2011).

Non‐aflatoxigenic *A. flavus* active ingredients of biocontrol products have traditionally been defined by VCG. VCA is used to track the active ingredients on crops, in the environment, and over seasons and to verify identity during manufacture (Atehnkeng et al., 2016; Cotty, 1994a, 1994b; Cotty et al., 2007; Ouadhene et al., 2023). *Aspergillus flavus* L strain populations are complex, with individual agricultural fields typically containing hundreds of VCGs (Barros et al., 2005; Bayman & Cotty, 1993). These VCGs diverged over millennia and during those periods mutation caused variability that can be detected at SSR loci both within and among VCGs (Grubisha & Cotty, 2010, 2015; Ortega‐Beltran & Cotty, 2018). Such mutations were detected in IT006, resulting in several closely related haplotypes belonging to that VCG (Table 4). VCGs used for biocontrol are selected so that all members of the VCG are atoxigenic. During evolution of these non‐aflatoxigenic VCGs, mutations accumulate in the 70 kb aflatoxin biosynthesis gene cluster causing multiple lesions that independently may result in loss of the ability to produce aflatoxins (Adhikari et al., 2016). The result of these aflatoxin gene cluster mutations is a highly stable non‐aflatoxigenic phenotype within the biocontrol VCGs (Adhikari et al., 2016). Parasexual recombination within VCGs increases the diversity of SSR haplotypes and contributes to the great diversity detected within *A. flavus* populations (Grubisha & Cotty, 2010; Leslie, 1993; Mehl et al., 2012; Papa, 1986). The diverse population structure of Southern European *A. flavus* populations is reflected in the high allelic diversity (Table 1) and high haplotypic diversity within the three studied countries (Table 2), similar to the diversity found in other reports utilizing these SSR markers to study *A. flavus* populations in the United States (Grubisha & Cotty, 2010) and Kenya (Islam et al., 2018). The high frequency of haplotypes belonging to VCG IT006 suggests that the biocontrol product AF‐X1 can be safely applied in Southern Europe without introducing an *A. flavus* VCG that is not naturally occurring. It also suggests that AF‐X1 is a readily available, ecologically safe tool for providing highly effective aflatoxin mitigation (Mauro et al., 2013, 2015, 2018).

*Aspergillus flavus* is a ubiquitous anamorphic fungal species that produces abundant asexual conidia on many organic substrates including material associated with several crops that are also susceptible to aflatoxin contamination (Klich, 2002; Ojiambo et al., 2018). However, several experimental studies suggest frequent sexual reproduction and its concomitant recombination (Horn et al., 2009, 2016; Moore et al., 2013). Recently Molo et al. (2019) and Molo et al. (2022) reported genetic exchange and sexual recombination when biocontrol strains of opposite mating types are used in the same formulation. They also reported possible sexual recombination in microplot trials with the two biocontrol products registered for use in the United States, AF36 and Afla‐Guard. In contrast, the data presented here show complete LD across the three countries (Figure S4). This indicates natural population structures in Greece, Spain and Serbia result predominantly from asexual reproduction. The inability to observe sexual recombination on the population level may be due to the rate at which the asexual conidia are produced (tens of thousands per day after colonization) versus the sexual ascospores, which according to Horn et al. (2009) number in the hundreds after months of development after sexual reproduction. Against the scope of natural variation seen in *A. flavus* populations, any sexual recombinants may be impossible to observe.

This clonal population structure has previously been reported for *A. flavus* populations in Kenya and Mexico where similar LD was measured (Islam et al., 2018; Ortega‐Beltran et al., 2020). Likewise, LD analyses showed no evidence of genetic exchange with other VCGs or sexual recombination for the VCG containing AF36, the first non‐aflatoxigenic *A. flavus* active ingredient used in the United States (Grubisha & Cotty, 2015). The VCG of AF36 is naturally distributed across North America (Ortega‐Beltran et al., 2016) in a manner similar to what is reported in the current study for IT006 with respect to the three countries reported here. AF36 has been widely utilized as a biocontrol agent in commercial agriculture in the United States since 1996 (Cotty et al., 2007) with no health or environmental ill effects, suggesting that widespread adoption of AF‐X1 to control aflatoxins across Southern Europe should also be safe and appropriate.

Two specific haplotypes, H76 and H588, had the greatest distribution across southern Europe but were only detected in Spain and Greece. High frequency of certain haplotypes can come about through two possibilities. First, the haplotype could be highly adapted to the environmental conditions found in the crop sampled. VCGs often contain significant haplotypic diversity (Grubisha & Cotty, 2010, 2015; Islam et al., 2021; Ortega‐Beltran et al., 2020), and any adaptive traits should be shared across all haplotypes within their respective VCGs due to parasexual recombination. In that scenario, many closely related haplotypes should be observed at high frequency, reflecting this shared adaptive success. The second possibility is that the observed high frequency of these two haplotypes could be due to rapid transient shifts in composition of *A. flavus* communities across a broad portion of southern Europe initiated by founder events (Ortega‐Beltran et al., 2020; Ortega‐Beltran & Cotty, 2018). Two such events have been described in North America in association with maize production in Sonora, Mexico and Louisiana, USA (Ortega‐Beltran & Cotty, 2018; Sweany et al., 2011). Although the founder events in Mexico were initially described using VCA, the population shift in Mexico was later shown to be caused by a single haplotype similar to what was observed in the current study with both H76 and H588 (Ortega‐Beltran et al., 2020).

Grain contamination with aflatoxins was detected in all three of the countries from which corn was collected. However, contamination was only detected in one of 2 years in both Spain and Serbia even though, among the three countries, corn produced in Serbia had the highest frequency and severity of contamination detected during the current study. These observations support prior work indicating production of corn with unacceptable aflatoxin concentrations in Southern Europe (Battilani et al., 2016; Gallo et al., 2012). Aflatoxin contamination varies widely between years and among fields and regions even within an individual country (Cotty et al., 2008). Like other plant disease problems, aflatoxin contamination requires in addition to the causal agent both a susceptible host and a conducive environment. Thus, it is difficult to relate populations of aflatoxin producers to frequencies of aflatoxin contamination without careful analysis of other predisposing factors (Cotty & Jaime‐Garcia, 2007; Giorni et al., 2016). The emphasis of the current study was on comparisons of populations of aflatoxin‐producing and closely related fungi. The samplings were not designed to compare aflatoxin contamination among countries and to identify possible mitigating factors. Future studies emphasizing comprehensive sampling and analysis of crops in Southern Europe tied with environmental analyses might provide insight on when and where investments in aflatoxin mitigation would be most profitable.

While the exact haplotype of MUCL54911, the strain which is the active ingredient in the biocontrol product AF‐X1, was not observed in any country studied here, close relatives were detected in each of the three countries (Figure S5). Testing of these related haplotypes using VCA showed that like MUCL54911, isolates with these closely related haplotypes belong to VCG IT006 (Table 4; Mauro et al., 2018). Since members of VCGs are clonally related (Atehnkeng et al., 2016; Grubisha & Cotty, 2010, 2015; Leslie, 1993), the presence of VCG IT006 in all three countries suggests it is endemic and well adapted to environments across this region. Combined, these observations suggest that AF‐X1 is an environmentally safe product that will likely be effective throughout Southern Europe.

## AUTHOR CONTRIBUTIONS

**Mohamed Ali Ouadhene:** Data curation (equal); formal analysis (equal); methodology (supporting); software (supporting); writing – original draft (lead). **Kenneth A. Callicot:** Data curation (equal); formal analysis (equal); methodology (equal); software (lead); writing – review and editing (equal). **Alejandro Ortega‐Beltran:** Data curation (supporting); formal analysis (supporting); resources (supporting); writing – review and editing (equal). **Hillary L. Mehl:** Resources (supporting); writing – review and editing (equal). **Peter J. Cotty:** Conceptualization (equal); methodology (supporting); supervision (supporting); writing – original draft (supporting); writing – review and editing (equal). **Paola Battilani:** Conceptualization (equal); funding acquisition (lead); methodology (supporting); project administration (lead); resources (lead); supervision (lead); writing – original draft (supporting); writing – review and editing (equal).

## CONFLICT OF INTEREST STATEMENT

The authors declare no conflicts of interest.

## Supporting information


**Figure S1.** Haplotype rarefaction curve for clone‐corrected data. GR, Greece; SB, Serbia; SP, Spain. Figure created using the ‘vegan’ package in R.
**Figure S2.** Principal coordinate analysis of *A. flavus* SSRs. Individual points represent individual haplotypes of *A. flavus* obtained from three different countries Greece (red, G‐Pop), Spain (green, Sp‐Pop) and Serbia (blue, Sb‐Pop). Principal coordinates 1 and 2 explain 68.6% and 13.95% of the genetic variation, respectively.
**Figure S3.** Measured variance (black lines) versus simulated variance for panmictic populations (histograms) for within population (A), among populations within countries (B), and among countries (C) strata.
**Figure S4.** Standardised index of association *r¯*_d_ as the measure of multilocus genotypic linkage disequilibrium (LD) in the clone‐corrected samples of *A. flavus* isolates from Greece (A), Spain (B) and Serbia (S). The dotted blue line indicates the calculated value for the actual data, while the histogram represents data from simulated recombining populations with the same allele frequencies.
**Figure S5.** Neighbour networks for each country generated by SplitsTree to showing the distance of all the haplotypes from each other and how they are close related to MUCL54911 the active ingredient of AF‐X1 in A. Greece, B. Spain and C. Serbia.

**Data S1.** Supporting Information.

## Data Availability

The authors declare that all data supporting findings of the study “Structure of Aspergillus flavus population associated with maize in Greece, Spain, and Serbia: implications for aflatoxin biocontrol on a regional scale” will be available upon the publication of the paper. The data contains informaation regarding sampling location, fungal population counting, aflatoxin analysis and SSR data.
