# Supplementary material for: Structure of Aspergillus flavus populations associated with maize in Greece, Spain, and Serbia: Implications for aflatoxin biocontrol on a regional scale
Source: Environ Microbiol Rep. 2024 Apr 17;16(2):e13249. doi: 10.1111/1758-2229.13249 (PMC11024511; doi:10.1111/1758-2229.13249)
Supplement: Supplementary file 1 — Figure S1. Haplotype rarefaction curve for clone‐corrected data. GR, Greece; SB, Serbia; SP, Spain. Figure created using the ‘vegan’ package in R. Figure S2. Principal coordinate analysis of A. flavus SSRs. Individual points represent individual haplotypes of A. flavus obtained from three different countries Greece (red, G‐Pop), Spain (green, Sp‐Pop) and Serbia (blue, Sb‐Pop). Principal coordinates 1 and 2 explain 68.6% and 13.95% of the genetic variation, respectively. Figure S3. Measured variance (black lines) versus simulated variance for panmictic populations (histograms) for within population (A), among populations within countries (B), and among countries (C) strata. Figure S4. Standardised index of association r¯ d as the measure of multilocus genotypic linkage disequilibrium (LD) in the clone‐corrected samples of A. flavus isolates from Greece (A), Spain (B) and Serbia (S). The dotted blue line indicates the calculated value for the actual data, while the histogram represents data from simulated recombining populations with the same allele frequencies. Figure S5. Neighbour networks for each country generated by SplitsTree to showing the distance of all the haplotypes from each other and how they are close related to MUCL54911 the active ingredient of AF‐X1 in A. Greece, B. Spain and C. Serbia. [file EMI4-16-e13249-s002.doc]

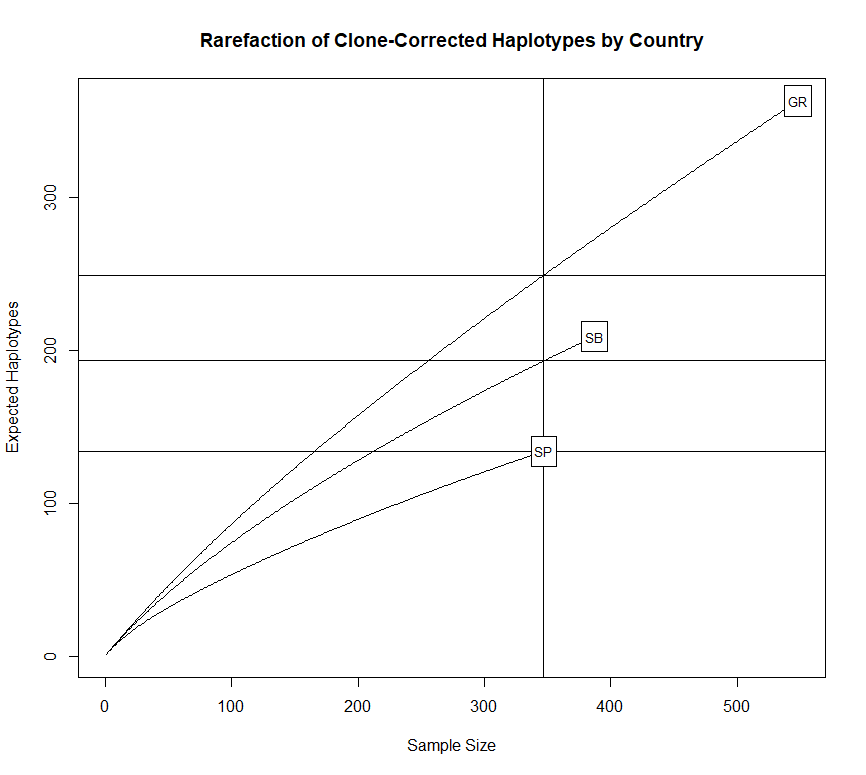


Figure S1. Haplotype rarefaction curve for clone-corrected data. GR= Greece. SB = Serbia. SP = Spain. Figure created using the “vegan” package in R.


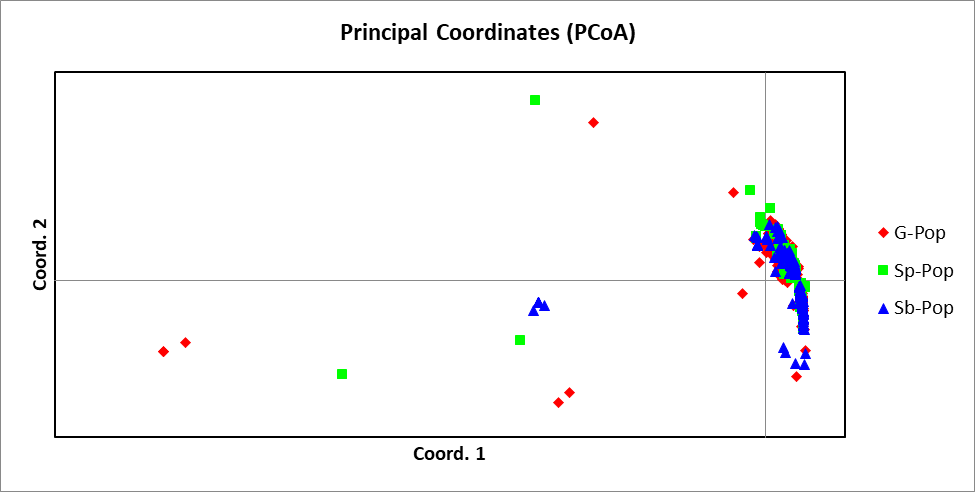


**Figure S2.** Principal coordinate analysis of A. flavus SSRs. Individual points represent individual haplotypes of A. flavus obtained from three different countries Greece (red, G-Pop), Spain (green, Sp-Pop) and Serbia (blue, Sb-Pop). Principal coordinates 1 and 2 explain 68.6% and 13.95% of the genetic variation, respectively.

**Figure S3.** Measured variance (black lines) versus simulated variance for panmictic populations (histograms) for within population (A), among populations within countries (B), and among countries (C) strata

Frequency


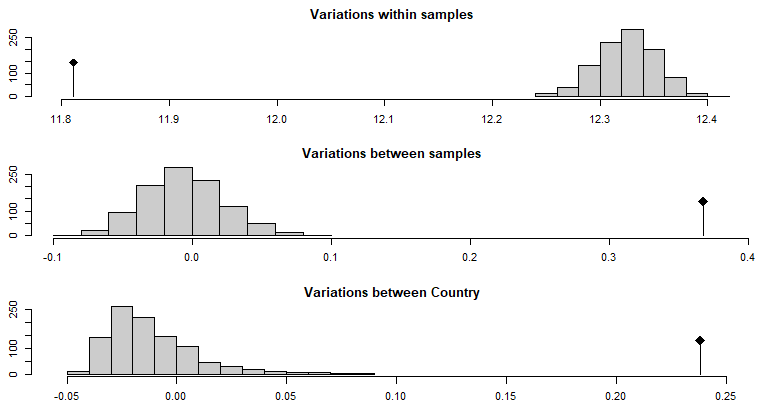

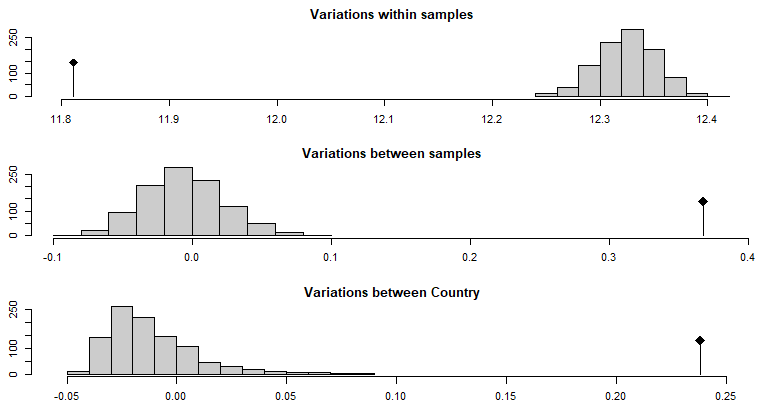


Frequency


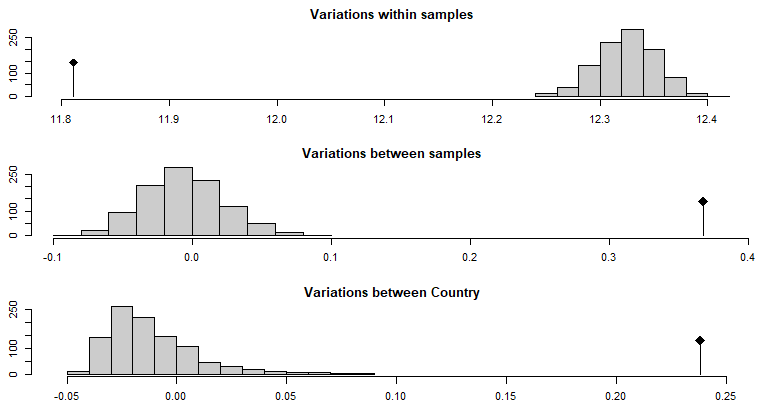


Frequency

Variance within populations

Variance among populations within countries

Variance among countries

A.

B.

C.


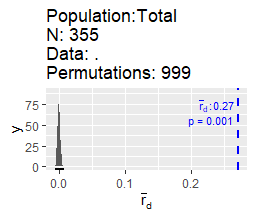


A


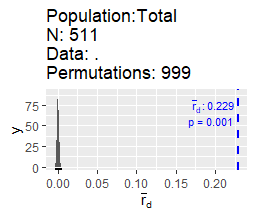

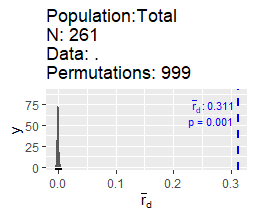


B

C

**Figure S4.** Standardized index of association r̅_d_ as the measure of multilocus genotypic linkage disequilibrium (LD) in the clone-corrected samples of *A.flavus* isolates from Greece (A), Spain (B) and Serbia (S). The dotted blue line indicates the calculated value for the actual data, while the histogram represents data from simulated recombining populations with the same allele frequencies.


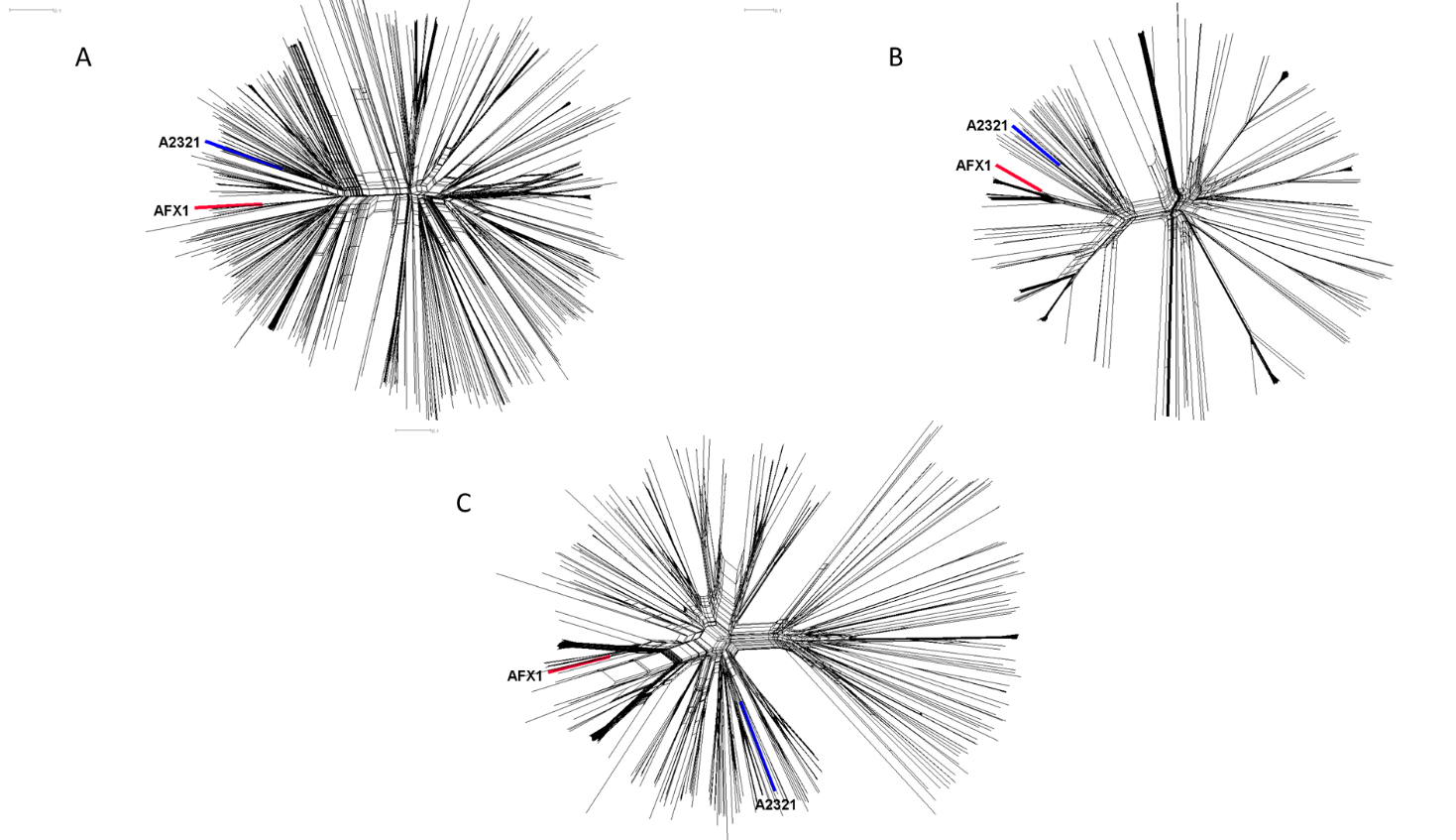


**Figure S5.** Neighbor networks for each country generated by SplitsTree to showing the distance of all the haplotypes from each other and how they are close related to MUCL54911 the active ingredient of AF-X1 in A. Greece, B. Spain and C. Serbia.
